# Crocin Improves Endothelial Mitochondrial Dysfunction *via* GPx1/ROS/KCa3.1 Signal Axis in Diabetes

**DOI:** 10.3389/fcell.2021.651434

**Published:** 2021-03-12

**Authors:** Xuemei Li, Yang Liu, Anqiang Cao, Chao Li, Luodan Wang, Qing Wu, Xinlei Li, Xiaohong Lv, Jiwei Zhu, Hua Chun, Ciren Laba, Xingchi Du, Yafang Zhang, Huike Yang

**Affiliations:** ^1^Department of Anatomy, Harbin Medical University, Harbin, China; ^2^Department of Anatomy, Heilongjiang University of Chinese Medicine, Harbin, China; ^3^Department of Cardiac Surgery, The Third People's Hospital of Chengdu, Institute of Cardiovascular Science, Chengdu, China; ^4^Department of Forensic Medicine, Harbin Medical University, Harbin, China; ^5^Department of Modern Medicine, Tibetan Traditional Medical College, Lhasa, China

**Keywords:** mitochondrial function, intermediate-conductance Ca^2+^ -activated K^+^ channels, endothelial dysfunction, crocin, diabetes mellitus

## Abstract

Mitochondrial dysfunction contributes to excessive reactive oxygen species (ROS) generation, which is a dramatic cause to promote endothelial dysfunction in diabetes. It was previously demonstrated that crocin protected the endothelium based on its diverse medicinal properties, but its effect on the mitochondrion and the potential mechanism are not fully understood. In this study, mitochondrial function was analyzed during the process of excessive ROS generation in high glucose (HG)-cultured human umbilical vein endothelial cells (HUVECs). The role played by KCa3.1 was further investigated by the inhibition and/or gene silence of KCa3.1 in this process. In addition, nicotinamide adenine dinucleotide phosphate (NADPH)-oxidase 2 (NOX2), superoxide dismutase 1 (SOD1), and glutathione peroxidase 1 (GPx1) were also detected in this study. Our data showed that crocin improved mitochondrial dysfunction and maintained normal mitochondrial morphology by enhancing the mitochondrial membrane potential (MMP), mitochondrial mass, and mitochondrial fusion. Furthermore, KCa3.1 was confirmed to be located in the mitochondrion, and the blockade and/or silencing of KCa3.1 improved mitochondrial dysfunction and reduced excessive ROS generation but did not affect NOX2 and/or the SOD1 system. Intriguingly, it was confirmed that KCa3.1 expression was elevated by ROS overproduction in the endothelium under HG and/or diabetes conditions, while crocin significantly suppressed this elevation by promoting GPx1 and subsequently eliminating ROS generation. In addition, crocin enhanced CD31, thrombomodulin (TM), and p-/t-endothelial nitric oxide synthase (eNOS) expressions as well as NO generation and decreased vascular tone. Hence, crocin improved mitochondrial dysfunction through inhibiting ROS-induced KCa3.1 overexpression in the endothelium, which in turn reduced more ROS generation and final endothelial dysfunction in diabetes.

## Introduction

Endothelial cells, viewed as a barrier structure between blood and vessel wall/tissues, function actively in maintaining the vascular homeostasis system under normal conditions (Favero et al., 2014). They are able to crucially regulate vascular tone, structure, and other various biological events by the production of a wide range of biological factors (Deanfield et al., 2007; Rajendran et al., 2013; McLaughlin et al., 2018). The diverse local environment elicits heterogeneous endothelial cell phenotypes and functions that respond to various pathological factors (Rajendran et al., 2013). Therefore, some harmful factors, such as hyperglycemia, excessive reactive oxygen species (ROS), oxidized low-density lipoprotein (LDL), cholesterol, etc., can cause damages to the endothelium and subsequent endothelial dysfunction. Endothelial dysfunction is prevalent in a variety of human disorders, including diabetic vascular complication, hypertension, chronic kidney failure, and so on (Rajendran et al., 2013; Widmer and Lerman, 2014). As a dominant source of cellular ROS besides the NOX system (Dan Dunn et al., 2015), mitochondria are generally injured by hyperglycemia, which results in mitochondrial dysfunction. Importantly, mitochondrial dysfunction is a dominant cause of more ROS production, leading to the development of endothelial dysfunction in diabetes (Widlansky and Hill, 2018).

As we all know, the endothelium-derived hyperpolarizing factor (EDHF) system is a vital endothelium-dependent vasodilator mechanism besides nitric oxide (NO) and prostacyclin (PGI2) (Grgic et al., 2009; Damkjaer et al., 2012). In this process, KCa3.1, a member of the Ca^2+^-activated K^+^ channel family, plays a crucial role in initializing EDHF–dilator responses by hyperpolarizing the endothelium in endothelium-dependent relaxation (Wolfle et al., 2009; Damkjaer et al., 2012). KCa3.1 dysfunction, as well as abnormal expression, is increasingly confirmed in diverse cardiovascular diseases (CVDs) associated with endothelial dysfunction (Feletou, 2009; Mathew John et al., 2018). In addition, growing evidence showed that KCa3.1 expression appears to be elevated in the kidneys of diabetic patients and mice (Huang et al., 2013, 2018). Moreover, KCa3.1 current and expression increased in diabetic serum-treated vascular smooth muscle cells (VSMCs) (Su et al., 2013). However, KCa3.1 expression profile and function have not been established in diabetic vascular endothelial cells. Intriguingly, besides the plasma membrane, KCa3.1 locates also in the mitochondrial inner membrane of tumor cells and has been proposed to potentially modulate mitochondrial function (Leanza et al., 2014; Klumpp et al., 2018). Therefore, we hypothesize that abnormal KCa3.1 might be closely associated with mitochondrial dysfunction, which subsequently contributes to excessive ROS generation and endothelial dysfunction in diabetes.

Crocin, the major pharmacological compounds of saffron, possesses potential antioxidant, anti-inflammatory, antiaging activities in the physical body (Ghorbanzadeh et al., 2016; Fagot et al., 2018; Liu et al., 2018). In the cardiovascular system, aside from its antioxidant and anti-inflammatory effects, crocin has been determined to dramatically improve vascular endothelial function, which is associated with KCa3.1 and NO signals in our previous study (Yang et al., 2018). Crocin induces KCa3.1 activation and subsequent NO generation in endothelial cells, which results in EDHF-/NO-type dilations in vessels (Yang et al., 2018). Therefore, these strongly imply that crocin contributes to the improvement of endothelial dysfunction, in which KCa3.1-regulated mitochondrial function vitally plays a role in diabetes.

## Materials and Methods

### Cell Culture

Human umbilical vein endothelial cells (HUVECs; ScienCell, USA) were cultivated in a special endothelial cell medium (ECM) containing high glucose (HG; 33 mmol/L) to establish the model of HG-induced endothelial dysfunction. In addition, this ECM was supplemented with 5% fetal bovine serum (FBS), 1% endothelial cell growth supplement (ECGS), and 1% penicillin/streptomycin solution (PS). HUVECs were cultivated in ECM with mannitol (33 mmol/L) and glucose (5.5 mmol/L) as control and normal, respectively. Besides crocin (Santa Cruz, USA), HUVECs were specially treated by KCa3.1 blocker {1-[(2-chlorophenyl)diphenylmethyl]-1H-pyrazole (TRAM-34)} and/or agonist (1-EBIO), aiming at confirming the roles of KCa3.1 in mitochondrial function and ROS-related protease activity. In addition, N-acetyl-L-cysteine (NAC, a ROS scavenger) and/or Rosup (an exogenous ROS donor) was also used to illustrate the effect of ROS on KCa3.1 expression. Passages 3–10 of HUVECs were used in this whole study.

### Experiment of Diabetes Model

According to previous studies (King, 2012; Xu et al., 2019), male Sprague–Dawley (SD) rats (180–220 g; Experimental Animal Center of China Pharmaceutical University) and male BALB/c mice (20–22 g; Experimental Animal Center of Harbin Medical University) were intraperitoneally injected with streptozotocin (STZ) (0.1 M, PH 4.5) at the dosage of 55 and 100 mg/kg body weight to induce diabetes mellitus model, respectively. Then, these animals were fed the high-sugar and high-fat diet (43.6% fat, 45.1% carbohydrate, and 11.3% protein). These rats and/or mice with fasting blood glucose levels >16.7 mmol/L were considered diabetic animals to use in this study. Mice and rats were mainly used in KCa3.1 expression profile analysis and myograph study of aortic rings, respectively.

Twenty diabetic mice were randomly divided into four groups (*n* = 5 for each group): three crocin groups (crocin-20,−40, and−60) and one diabetic mellitus control group (DM). Crocin was administered once daily by intraperitoneal injection at the dosage of 20, 40, and 60 mg/kg body weight for 4~6 weeks in all crocin groups. An equal volume of 0.9% saline solution daily was used in the DM group. Five normal mice were used as normal control. Body weight and fasting blood glucose levels of mice in all groups were detected in this experiment. After the treatment, aorta tissue samples were collected for immunofluorescence staining. Moreover, 60 SD rats were divided into two groups (*n* = 30 for each group): normal (Norm) and DM groups. Aortas in each group were rapidly excised for the next myograph study after euthanasia of rats.

### Myograph Study

Aortic rings (3-mm length) of 30 SD rats were prepared and the tension was measured using a myograph system (Xinhang JZ101, Beijing) in Krebs buffer bubbled continuously with 95% O_2_ and 5% CO_2_ at 37°C, as described in the research of Yang et al. (2018). α-Receptor agonist phenylephrine (PE; 10^−5^ mol/L)-precontracted aortic rings were evaluated in the absence or presence of acetylcholine (ACh; 10^−10^-10^−6^ mol/L) and/or crocin (10^−9^-10^−5^ mol/L). In a portion of this research, aortic rings were pretreated with crocin (10^−5^ mol/L) for 30 min, and in turn the contractive response to PE (10^−9^-10^−5^ mol/L) was tested.

### Transmission Electron Microscope Investigation

Cells were scraped gently and centrifuged for 5 min at 1,000 rpm, which was followed by fixation with glutaraldehyde (2.5%) overnight and subsequent O_S_O_4_ (2%) for 2 h. In turn, graded alcohol was used to dehydrate the samples before embedding with Epon 812. Ultrathin sections of the samples were stained with uranium acetate and lead citrate, aiming at the observation under a transmission electron microscope (Hitachi, Tokyo, Japan).

### Small Interfering RNA Transfection

Three different small interfering RNA (siRNA) sequences (siRNA-1,−2, and−3) to target KCa3.1 gene (RiboBio Co., Ltd., China) were designed to knock down KCa3.1, and the optimal siRNA was used in this study. KCa3.1–siRNA (100 nmol/L) was transfected into HUVECs with a 50–75% confluence by the riboFECT™ CP regent following the manufacturer's protocol. The cells were treated with crocin for 48–72 h after siRNA transfection. The scramble siRNA was used as a negative control (NC).

### Western Blotting Analysis

The whole protein extracts were measured by bicinchoninic acid (BCA) assay (Pierce, Rockford, IL, USA) following the manufacturer's protocol. Proteins were then separated by 10–15% sodium dodecyl sulfate (SDS)–polyacrylamide gel electrophoresis (PAGE) and transferred to nitrocellulose filter or polyvinylidene fluoride (PVDF) membrane. After blocking with 5% milk, the membrane was probed with primary antibodies, including KCa3.1, p-endothelial nitric oxide synthase (eNOS), t-eNOS, CD31, thrombomodulin (TM), NADPH oxidase 2 (NOX2), superoxide dismutase 1 (SOD1), glutathione peroxidase 1 (GPx1), 4-hydroxynonenal (4HNE), β-actin, and glyceraldehyde 3-phosphate dehydrogenase (GAPDH) overnight at 4°C. In turn, the membrane was incubated with horseradish peroxidase (HRP)-conjugated secondary antibody for 1 h at room temperature and then developed using an enhanced chemiluminescence (ECL) detection system (Amersham Pharmacia Biotech). All experiments were performed at least in triplicate and analyzed using Quantity-One software 4.6.2 (Bio-Rad).

### Measurement of Intracellular Reactive Oxygen Species

Evaluation of ROS was performed by using 2′,7′-dichlorofluorescein diacetate (DCFH-DA; Beyotime, China), a fluorescent probe of ROS. Concisely, after diverse treatments such as crocin, Rosup, inhibitors, and siRNA, the HG-cultured HUVECs were incubated with DCFH-DA (100 μmol/L) at 37°C for 30 min. Images were obtained using a fluorescence microscope (Olympus-BX51, Japan). In obtained images, the fluorescence intensity was analyzed through calculating the mean optical density (MOD) using Image Pro-Plus 6.0 software referring to the previous method (Yang et al., 2018).

### Glutathione Peroxidase Activity Assay

In the present study, total glutathione peroxidase (GPx) Assay Kit (Beyotime, China) was used to detect the effect of crocin on GPx activity according to the manufacturer's instruction. This assay is a coupled reaction system that the oxidation of reduced glutathione accompanies NADPH oxidation or NADP^+^ generation, which is catalyzed by glutathione reductase. One unit of GPx activity was considered the oxidation of 1 μmol of NADPH to NADP^+^ in 1 min at 25°C, pH 8.0. The decrease in NADPH was determined colorimetrically at 340 nm during the oxidation of NADPH to NADP^+^ using an absorbance microplate reader (Molecular Devices, M2).

### Mitochondrial Membrane Potential Detection

According to the manufacturer's instructions, mitochondrial membrane potential (MMP) was probed using a cationic dye JC-1 (5,5′,6,6′-tetrachloro-1,1′,3,3′-tetraethyl–imidacarbocyanine iodide) (Beyotime, China). When MMP remains at a high level, JC-1 can be driven into mitochondrial matrix to form JC-1 aggregate, which shows red fluorescence at 585 nm (absorption)/590 nm (emission). Oppositely, lower MMP leads to dominant JC-1 monomer formation, presenting green fluorescence at 485 nm (absorption)/535 nm (emission). In the present study, the MMP was analyzed through the ratio of red/green fluorescence intensity by flow cytometry (Becton Coulter EPICS XL, USA) and images by a fluorescence microscope.

### Determination of Nitric Oxide Production

Fluorescence probe 4-amino-5-methylamino-2,7-difluorofluorescein diacetate (DAF-FM DA) (Beyotime, China) was used to detect NO as described previously (Chung et al., 2012; Yang et al., 2018). Briefly, the HG-cultured HUVECs were incubated with DAF-FM DA (5 μmol/L) for 30 min at 37°C prior to crocin treatment. Image was acquired using a fluorescence microscope, and the fluorescence intensity of DAF-FM DA was assessed as described above for ROS measurement.

### Intracellular Ca^2+^ Measurement

Following the previous protocol (Yang et al., 2018), the intracellular Ca^2+^ was probed using Fluo-3 AM (Beyotime, China) and measured *in situ* by confocal microscopy (Nikon, ECLIPSE Ti-U, Japan). Briefly, the HG-cultured HUVECs were treated with crocin for at least 48 h and in turn incubated in serum-free ECM containing Fluo-3 AM (25 μmol/L) for 30 min at 37°C. The cells were then rinsed using Hanks' balanced salt solution (HBSS) to remove excess dye and detected by confocal microscopy. The fluorescence intensity of Fluo-3 AM was assessed as described above for ROS and NO measurements.

### Mitochondrial Mass and KCa3.1 Immunofluorescence Tests

In this study, mitochondria were labeled using Mito-Tracker Red CMXRos (Beyotime, China), a mitochondria marker, to assess mitochondrial mass. Concisely, HUVECs were seeded into the 2-μg/cm^2^ fibronectin-coated culture dish and subjected to the specified pretreatments such as crocin, KCa3.1 agonists, and/or inhibitors. According to the manufacturer's protocol, 200 nM Mito-Tracker Red CMXRos was added to incubate HUVECs for 30 min at 37°C. In turn, Mito-Tracker-labeled HUVECs were probed using the primary antibody KCa3.1 at 4°C overnight after the paraformaldehyde (4%) fixation. And then, the cells were incubated using Alexa Fluor 488-labeled secondary antibody for 1 h at 37°C after the 5% goat serum blocking, and the nuclei were labeled by 4′,6-diamidino-2-phenylindole (DAPI). Confocal microscopy was used to take the images.

Aorta ring tissues from diabetic mice were fixed with 4% paraformaldehyde, embedded in optimum cutting temperature (OCT) compound, and cryosectioned into 6-mm-thick sections. Referring to our previous methods (Yang et al., 2013, 2018), an immunofluorescence test of KCa3.1 was performed on the prepared sections as described above.

### Antibodies

Antibodies include anti-KCa3.1 (Abcam, ab229593, and Alomone Labs, #ALM-051), anti-p-eNOS (Cell Signaling Technology, #9571s), t-eNOS (Cell Signaling Technology, #9572s), anti-TM (Proteintech, 14318-1-AP), anti-NOX2 (Proteintech, 19013-1-AP), anti-SOD1 (Proteintech, 10269-1-AP), anti-CD31 (Proteintech, 66065-1-Ig), GPx1 (Cell Signaling Technology, #3206S), anti-4HNE (Abcam, ab46545), anti-β-actin (Santa Cruz, sc-47778), and anti-GAPDH (Santa Cruz, sc-47724).

### Statistics

Data are analyzed using GraphPad Prism 5.0 (GraphPad Software Inc., USA) and presented as the mean ± SEM of at least three experiments. Student's *t*-test and one-way analysis of variance (ANOVA) were performed for statistical analysis. Differences with *p*-values ≤ 0.05 were considered statistically significant.

## Results

### Crocin Improves Mitochondrial Function in High Glucose-Cultured Endothelial Cells

**Figure 1:**
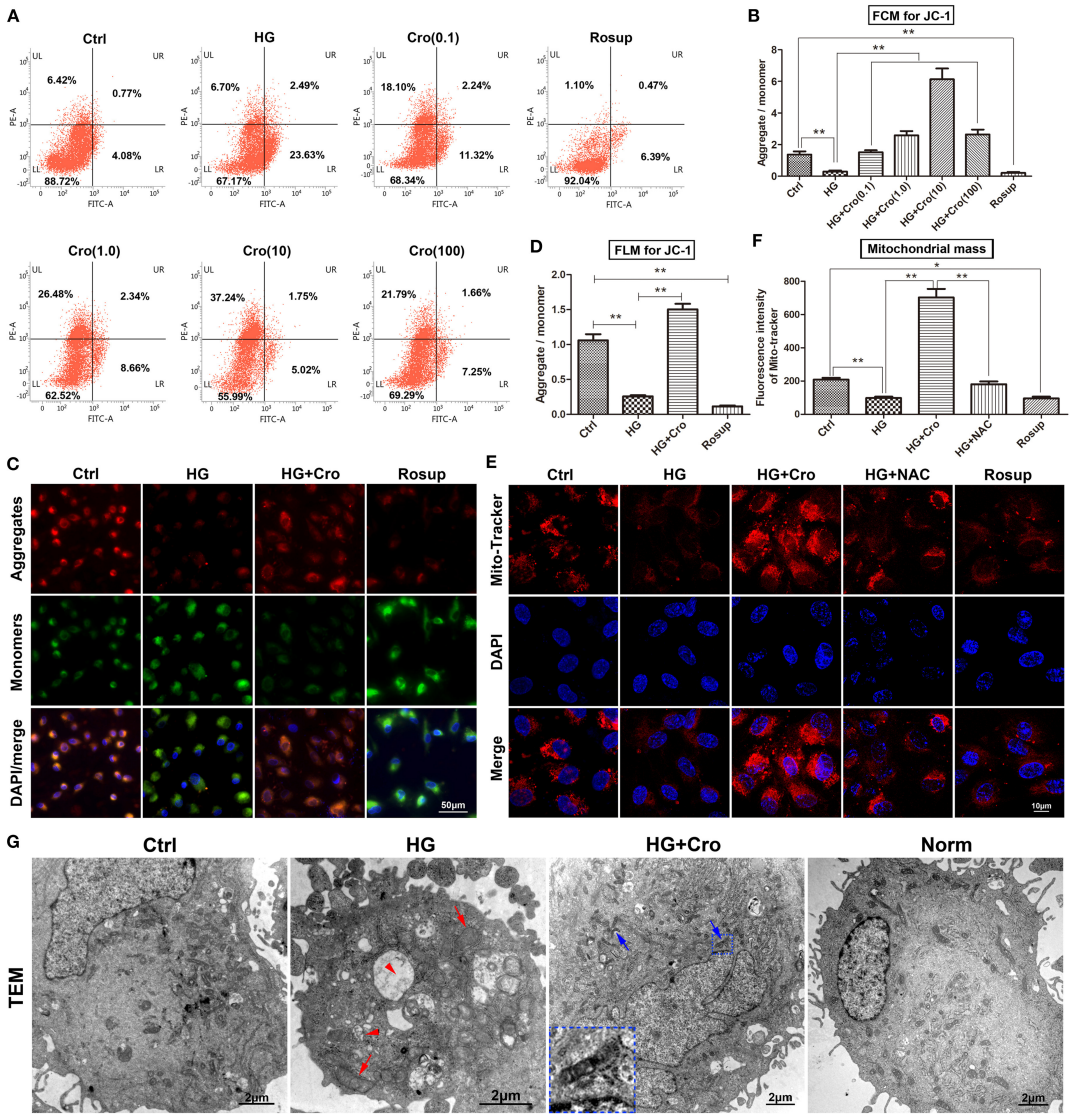
Crocin improved mitochondrial function in HG-cultured endothelial cells. The MMP and mitochondria mass were detected to assess the mitochondrial function. **(A,B)** The ratio of aggregates (red)/monomers (green) was analyzed to assess the MMP by flow cytometry in JC-1-probed cells. Data confirmed that HG dissipated the MMP, which was reversed by crocin in a dose-dependent fashion (0.1, 1, 10, and 100 μmol/L). **(C,D)** Fluorescence microscopy showed the same result of lower aggregates (red) and higher monomers (green) in both HG and Rosup groups compared with the control and crocin (10 μmol/L) groups. **(E,F)** Mitochondrial marker MitoTracker Red indicated that HG decreased the mitochondrial mass, which in turn was significantly reversed by crocin. Here, Rosup, as a positive control, also strongly reduced the MMP and mitochondrial mass in this experiment. **(G)** TEM images illustrated that HG induced serious apoptosis and mitochondrial damage, including fragmented cristae, swelling (red arrow), and vacuoles (red arrow head) compared to the control and normal groups. However, abundant unbroken mitochondria and even some fusing mitochondria (blue arrow) were shown in the crocin group compared to the HG group, and the small inset in this panel shows an amplified image of mitochondrial fusion. Ctrl, control (mannitol 33 mmol/L); HG, high glucose; Cro, crocin; NAC, N-acetyl-L-cysteine; FCM, flow cytometry; FLM, fluorescence microscopy; TEM, transmission electron microscope; Norm, normal glucose level; MMP, mitochondrial membrane potential. Results are shown as means ± SEM of three independent experiments. ^*^p < 0.05, ^**^p < 0.01. One-way ANOVA followed by Tukey's multiple comparisons test.

Given the dominant source of cellular ROS, mitochondria are considered a possible target of crocin for the suppression of ROS generation. In this study, the MMP, mitochondrial mass, and mitochondrial morphology were detected to assess mitochondrial function. Based on the ratio of aggregates/monomers, JC-1 probe data from both flow cytometry and fluorescence microscopy confirmed that, to a great extent, HG dissipated the MMP of endothelial cells compared to the control group, whereas crocin (0.1–100 μmol/L) could significantly restore the MMP with a peak at 10 μmol/L (Figures 1A–D). Additionally, the mitochondrial marker MitoTracker Red indicated that HG decreased the mitochondrial mass compared to the control, which, in turn, was significantly reversed by crocin (10 μmol/L) (Figures 1E,F). Similar to crocin, NAC (10 μmol/L) elevated the mitochondrial mass under HG conditions (Figures 1E,F). Rosup, as a positive control, also strongly reduced the MMP and mitochondrial mass in this experiment similar to HG (Figures 1A–F). In addition, representative TEM images illustrated that HG induced serious apoptosis and mitochondrial damage compared to the control and/or normal groups (Figure 1G). These destroyed mitochondria were characterized by fragmented cristae, swelling, and vacuoles in the HG group (Figure 1G). Unlike HG, crocin could significantly protect against the destruction of mitochondria induced by hyperglycemia. As shown in Figure 1G, large amounts of unbroken mitochondria were distributed in the cytoplasm of the crocin group, among which some fusing mitochondria were also captured by TEM (Figure 1G).

### Effect of KCa3.1 on Mitochondrial Function in High Glucose-Cultured Endothelial Cells

**Figure 2:**
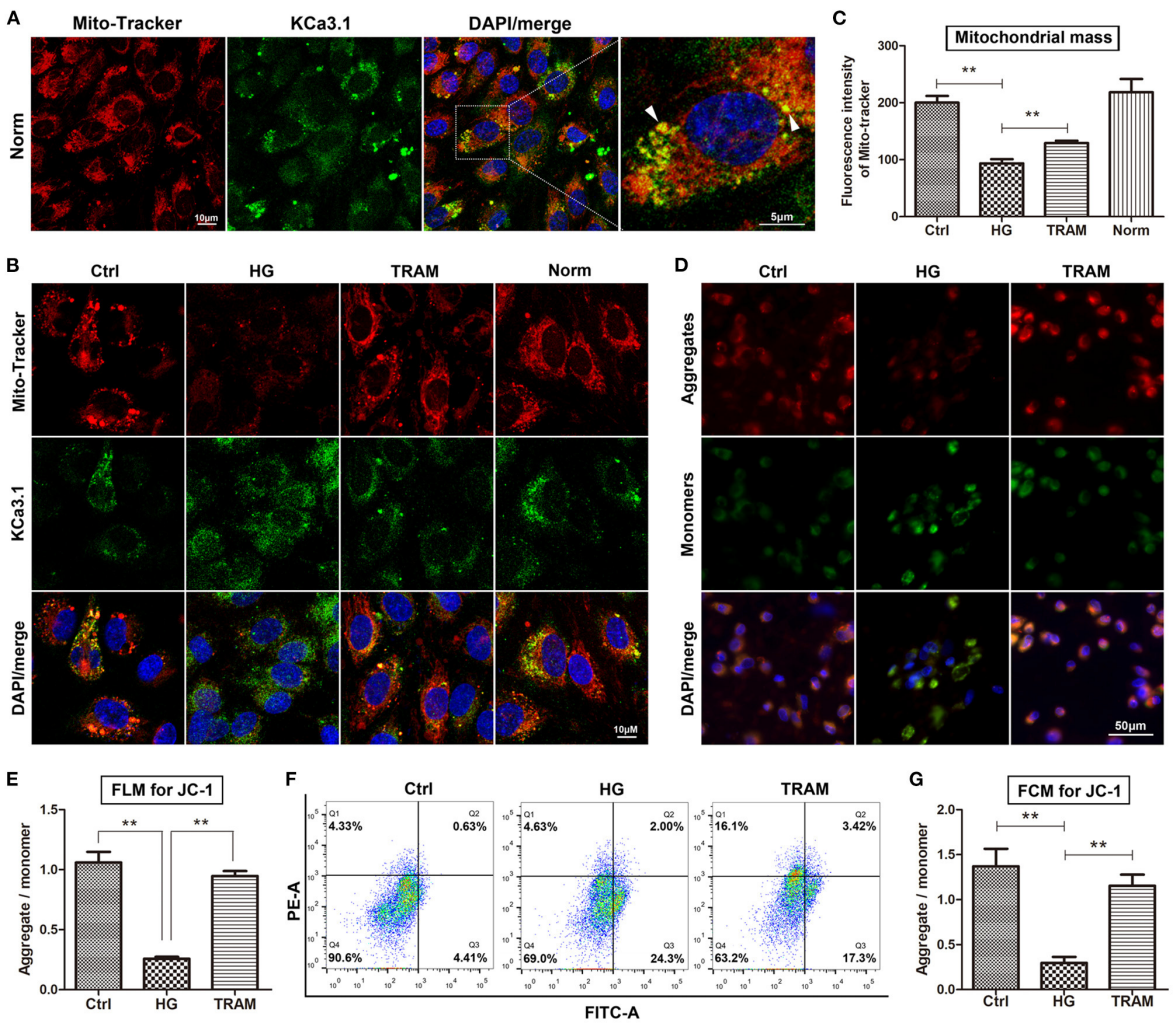
Effect of KCa3.1 on mitochondrial function in HG-cultured endothelial cells. **(A)** Confocal microscopy confirmed that KCa3.1 colocalized with MitoTracker Red in the mitochondria (arrow head). **(B,C)** Confocal images showed that KCa3.1 blocker TRAM-34 (10 μmol/L) remarkably increased mitochondrial mass compared to HG. **(D–G)** Flow cytometry and immunofluorescence staining assays indicated that TRAM-34 significantly improved the lower MMP induced by HG in JC-1-probed cells. Ctrl, control (mannitol 33 mmol/L); HG, high glucose; TRAM, TRAM-34 {1-[(2-chlorophenyl)diphenylmethyl]-1H-pyrazole}; Norm, normal glucose level; FCM, flow cytometry; FLM, fluorescence microscopy; MMP, mitochondrial membrane potential. Results are presented as means ± SEM of at least three independent experiments. ^**^p < 0.01. One-way ANOVA followed by Tukey's multiple comparisons test.

Our research clearly confirmed that KCa3.1 colocalized with MitoTracker Red in the mitochondria according to confocal microscopy (Figure 2A). Considering its possible relevance to mitochondrial function, we determined the regulatory effect of KCa3.1 on mitochondrial mass and the MMP using the KCa3.1 blocker TRAM-34 (10 μmol/L; Sigma, USA). Confocal microscopy showed that the mitochondrial mass increased remarkably in the TRAM-34 treatment group compared to the HG group, which showed levels similar to those in the control and/or normal (Figures 2B,C). Additional data from the JC-1 assay revealed that the blockade of KCa3.1 channels could significantly improve the HG-induced lower MMP according to flow cytometry and fluorescence microscopy (Figures 2D–G). These results suggested that KCa3.1 in mitochondria potentially controlled mitochondrial function and in turn mitochondrial ROS generation.

### Effect of KCa3.1 on Reactive Oxygen Species Production and Superoxide Dismutase 1, NADPH Oxidase 2, Glutathione Peroxidase 1, and 4-Hydroxynonenal in High Glucose-Cultured Endothelial Cells

**Figure 3:**
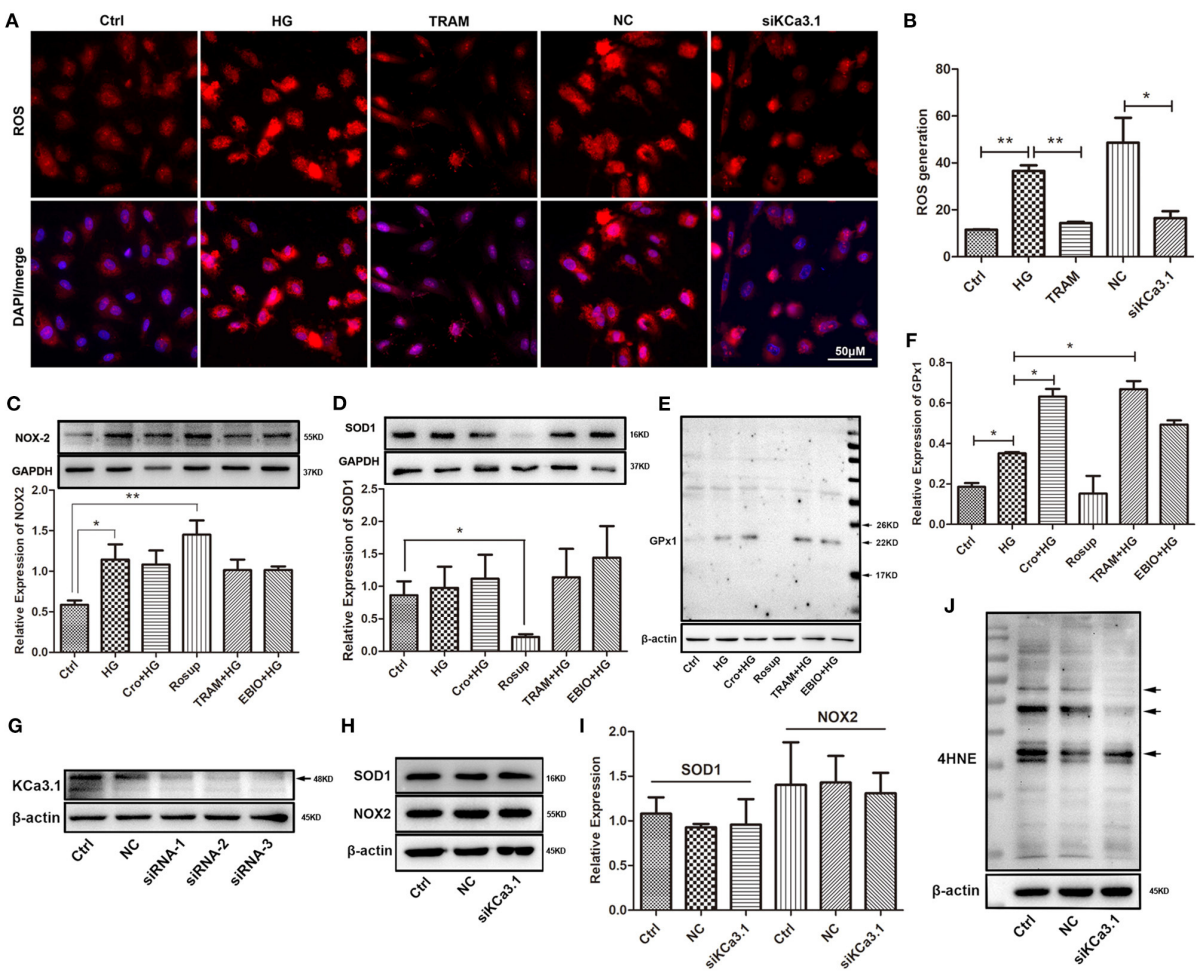
Effect of KCa3.1 on ROS production, SOD1, NOX2, GPx1, and 4HNE in HG-cultured endothelial cells. **(A,B)** Immunofluorescence staining identified that both blockade and knockdown of KCa3.1 significantly reduced the overproduction of ROS compared to the HG and negative control groups. **(C,D)** For KCa3.1, the blockade by TRAM-34 (10 μmol/L) and activation by 1-EBIO (10 μmol/L) had no alteration on NOX2 and SOD1 expressions. Moreover, Rosup enhanced NOX2 expression and reduced SOD1 expression, respectively. **(E,F)** GPx1 expression was slightly enhanced by HG compared to the control group, and the blockade of KCa3.1 dramatically increased GPx1 expression compared with HG. Rosup prominently suppressed GPx1 expression compared to the control group. **(G)** Western blotting showed that KCa3.1 was knocked down by three siRNAs of KCa3.1 (siRNA-1,−2, and−3). **(H–J)** The silencing of KCa3.1 could not alter the NOX2 and SOD1 expression, but it suppressed the 4HNE production (arrow). Ctrl, control (mannitol 33 mmol/L); HG, high glucose; Cro, crocin; TRAM, TRAM-34 {1-[(2-chlorophenyl)diphenylmethyl]-1H-pyrazole}; EBIO, 1- EBIO; NC, negative control; siKCa3.1, siRNA of KCa3.1; SOD1, superoxide dismutase 1; NOX2, NADPH oxidase 2; GPx1, glutathione peroxidase 1; 4HNE, 4-hydroxynonenal. Results are presented as means ± SEM of at least three independent experiments. ^*^p < 0.05, ^**^p < 0.01. One-way ANOVA followed by Tukey's multiple comparisons test.

To further confirm the possible effect of KCa3.1 on ROS, we tested ROS production after both blockade and silencing of KCa3.1. Research indicated that the blockade of KCa3.1 and/or knockdown of KCa3.1 significantly reduced this increase in ROS induced by HG (Figures 3A,B). To further characterize the possible mechanism of KCa3.1 action against HG-induced ROS production, the expression of NOX2, SOD1, and GPx1 was detected by Western blotting. Data indicated that the blockade and/or activation of KCa3.1 had no influence on NOX2 and SOD1 expression after the administration of TRAM-34 and/or 1-EBIO (10 μmol/L, Abcam, USA), respectively (Figures 3C,D). In addition, Western blotting revealed that KCa3.1 was significantly knocked down by three different siRNAs (siRNA-1,−2, and−3), respectively (Figure 3G). A similar result showed that the silencing of KCa3.1 could not alter NOX2 and SOD1 expression, as shown in Figures 3H,I. Additionally, KCa3.1 deficiency could suppress the production of 4HNE originating from redox reactions under hyperglycemia (Figure 3J). These data suggested that KCa3.1 could principally target mitochondria rather than the NOX2 and SOD1 systems to modulate ROS generation and subsequent peroxidation products. Moreover, GPx1 expression was significantly increased by the blockade of KCa3.1 (Figures 3E,F), suggesting that GPx1 might be a potential regulator in this process.

### Crocin Suppresses the Upregulation of Endothelial KCa3.1 Induced by Hyperglycemia *in vitro* and *in vivo*

**Figure 4:**
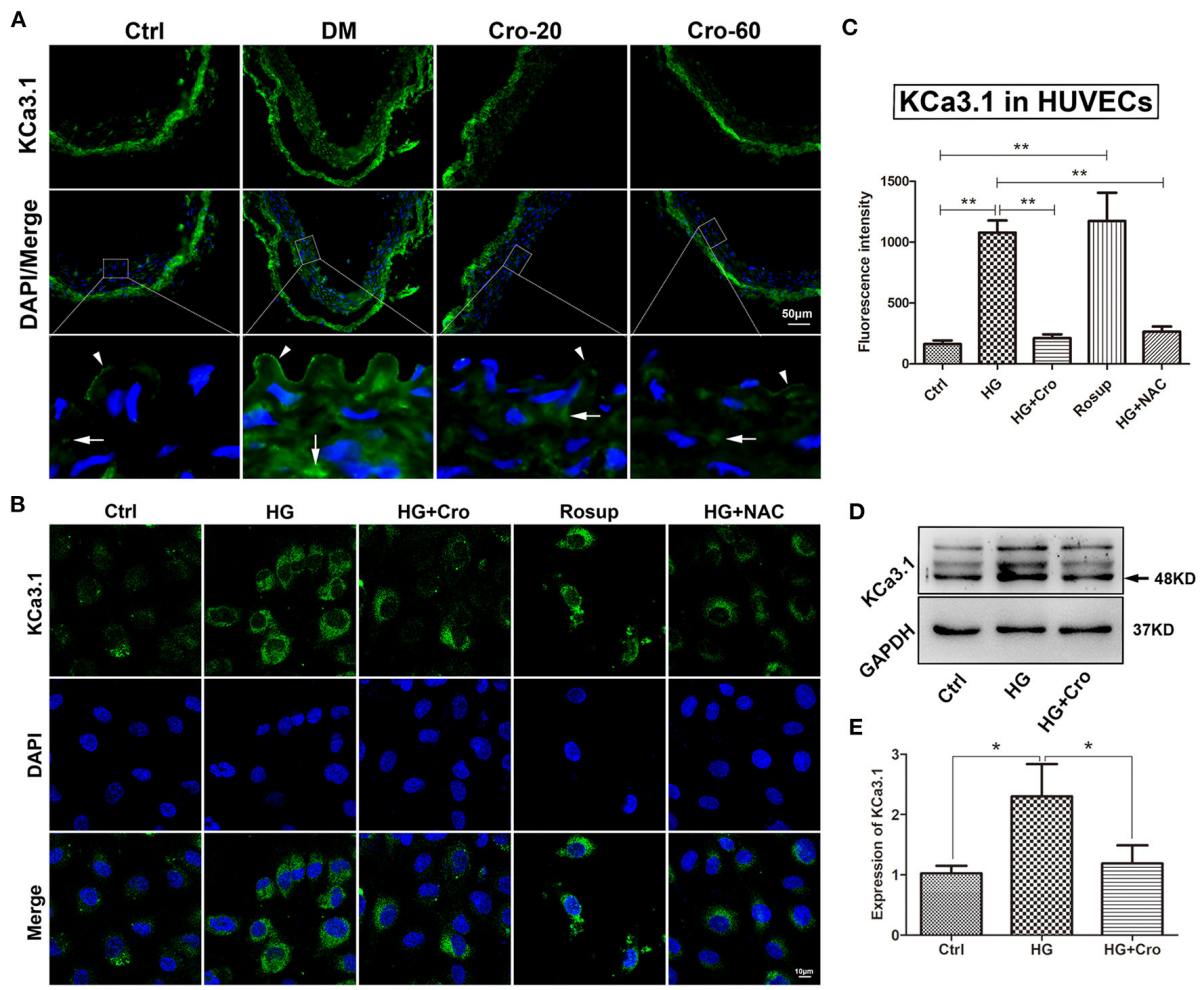
Crocin suppresses the upregulation of endothelial KCa3.1 induced by hyperglycemia. KCa3.1 expression profiles were explored in an aortic artery of diabetic mice and HG-cultured HUVECs to evaluate the regulatory role of crocin. **(A)** Immunofluorescence staining revealed KCa3.1 expression was significantly elevated in endothelial cells (arrow head) and vascular smooth muscle cells (arrow) in diabetes mellitus group (DM) compared to the control group (Ctrl), whereas its expression decreased in both crocin treatment groups. **(B,C)** Confocal images indicated that KCa3.1 was upregulated by HG in HUVECs, which was reduced by crocin (10 μmol/L) and NAC (10 μmol/L), respectively. Rosup (125 μg/ml), a positive control generating exogenous ROS, prominently enhanced the KCa3.1 expression in HUVECs. **(D,E)** Western blotting showed that crocin decreased the increased KCa3.1 induced by HG in HUVECs. DM, diabetes mellitus; Cro-20,−60, crocin 20, 60 mg/kg; Ctrl, control; Cro, crocin; HG, high glucose; NAC, N-acetyl-L-cysteine; HUVECs, human umbilical vein endothelial cells; ROS, reactive oxygen species. Results are shown as means ± SEM of three independent experiments. ^*^p < 0.05, ^**^p < 0.01. One-way ANOVA followed by Tukey's multiple comparisons test.

In the present study, KCa3.1 expression profiles were explored in the artery endothelium of diabetic rats and HG-cultured HUVECs. Our data demonstrated that the expression of KCa3.1 was significantly elevated by hyperglycemia in the arterial endothelium and/or by HG in HUVECs (Figure 4). Specifically, KCa3.1 expression was significantly increased in vascular endothelial and smooth muscle cells of the diabetic artery compared with those of the normal artery (Figure 4A). Further results showed that Rosup (25–150 μg/ml) prominently enhanced KCa3.1 expression, with a peak at 125 μg/ml (data not shown). Hence, the dosage of 125 μg/ml was used optimally in the following studies. Furthermore, NAC inhibited this upregulation of KCa3.1 expression induced by HG (Figures 4B,C). These data revealed that hyperglycemia-induced ROS was a key cause of elevated KCa3.1 expression in diabetes mellitus. Furthermore, the effect of crocin on KCa3.1 expression upregulation induced by hyperglycemia and/or HG was detected in this study. Interestingly, 20, 40 (data not shown), and 60 mg/kg crocin significantly counteracted the upregulation of KCa3.1 expression *in vivo* (Figure 4A). Additionally, the same results were supported after the application of crocin (10 μmol/L) during *in vitro* studies (Figures 4B–D).

### Effect of Crocin on Reactive Oxygen Species Production, Superoxide Dismutase 1, NADPH Oxidase 2, Glutathione Peroxidase 1, and Intracellular Ca^2+^ in High Glucose-Cultured Endothelial Cells

**Figure 5:**
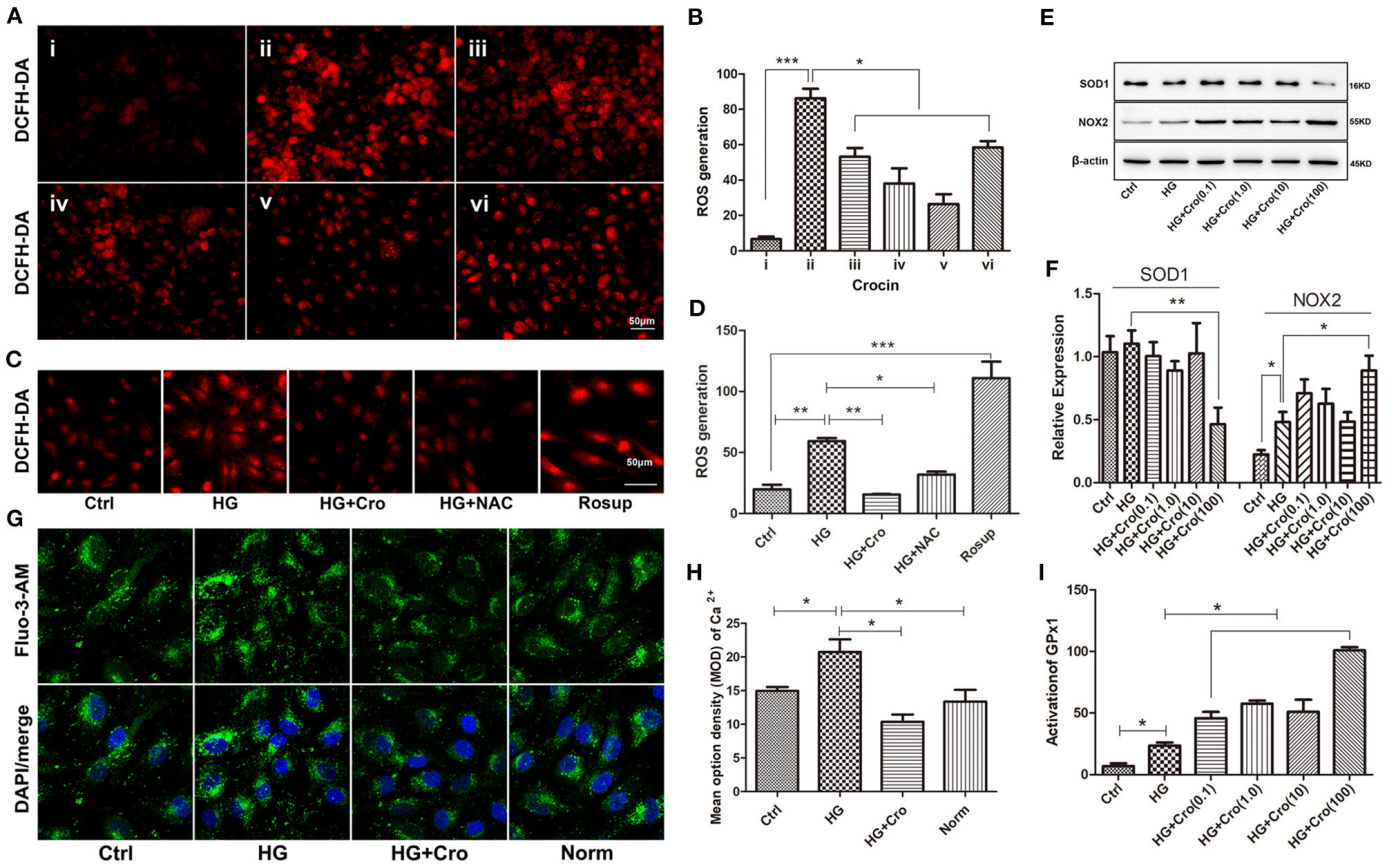
Effect of crocin on ROS production, SOD1, NOX2, and GPx1 in HG-cultured endothelial cells. **(A,B)** ROS fluorescent probe DCFH-DA revealed that HG induced excessive ROS production, whereas crocin significantly counteracted this increase in the dose range of 1–10 μmol/L. i: control (mannitol 33 mmol/L), ii–vi: HG (33 mmol/L) + crocin (0, 0.1, 1, 10, and 100 μmol/L). **(C,D)** Both crocin (10 μmol/L) and NAC (10 μmol/L) reduced the HG-induced increase of ROS in HUVECs. Rosup (125 μg/ml) was used as a positive control to generate excessive ROS in this experiment. **(E,F)** Western blotting showed that HG has an enhanced effect on NOX2 expression rather than SOD1. Crocin (0.1–10 μmol/L) did not alter the expression of NOX2 and SOD1 in HG-cultured endothelial cells; however, crocin at 100 μmol/L reduced SOD1 expression and enhanced NOX2 expression, respectively. **(G,H)** The confocal microscope showed that HG elevated intracellular Ca^2+^ probed by Fluo-3 AM compared to the control and normal groups, but crocin (10 μmol/L) reversed the increased intracellular Ca^2+^ concentration that is close to normal levels. **(I)** GPx activity assay revealed that HG increased GPx1 activation compared to the control group, and GPx1 is also promoted by crocin in the gradient dose (0.1, 1, 10, and 100 μmol/L). Ctrl, control (mannitol 33 mmol/L); HG, high glucose; Cro, crocin; NAC, N-acetyl-L-cysteine; Norm, normal glucose level; HUVECs, human umbilical vein endothelial cells; ROS, reactive oxygen species; SOD1, superoxide dismutase 1; NOX2, NADPH oxidase 2; GPx1, glutathione peroxidase 1. Results are presented as means ± SEM of at least three independent experiments. ^*^p < 0.05, ^**^p < 0.01, ^***^ *p* < 0.001. One-way ANOVA followed by Tukey's multiple comparisons test.

Furthermore, the effects of crocin on ROS generation and SOD1, NOX2, GPx1, and Ca^2+^ were analyzed in HG-cultured endothelial cells. Immunofluorescence assays revealed that crocin (0.1–100 μmol/L) could significantly counteract the increase in ROS induced by HG, which reached a maximum effect at 10 μmol/L (Figures 5A,B). In contrast to the control, Rosup induced excessive ROS generation, similar to HG (Figures 5C,D). Additionally, in parallel with crocin (10 μmol/L), NAC suppressed the excessive ROS generation induced by HG (Figures 5C,D). Further findings indicated that crocin enhanced the activation of GPx1 in a dose-dependent manner (Figure 5I). For NOX2 and SOD1, crocin did not significantly affect their expression in the low dose range of 0.1–10 μmol/L but enhanced NOX2 expression and reduced SOD1 expression at the high concentration of 100 μmol/L (Figures 5E,F). These data strongly implied that the GPx1 enzyme, but not NOX2 and SOD1, might be another modulatory target of crocin (10 μmol/L) to reduce excessive ROS generation in HG-cultured endothelial cells. As shown in previous studies, KCa3.1-controlled membrane hyperpolarization could provide a driving force for Ca^2+^ influx (Yang et al., 2014, 2018). Based on our prior data on hyperglycemia-elevated KCa3.1 expression, it appears reasonable to assume that HG might enhance the intracellular Ca^2+^ in endothelial cells. Our subsequent study confirmed that intracellular Ca^2+^ increased in HG-cultured HUVECs compared with the control and/or normal group and was significantly repressed by crocin (Figures 5G,H).

### Crocin Improves Hyperglycemia-Induced Endothelial Dysfunction *in vitro* and *in vivo*

**Figure 6:**
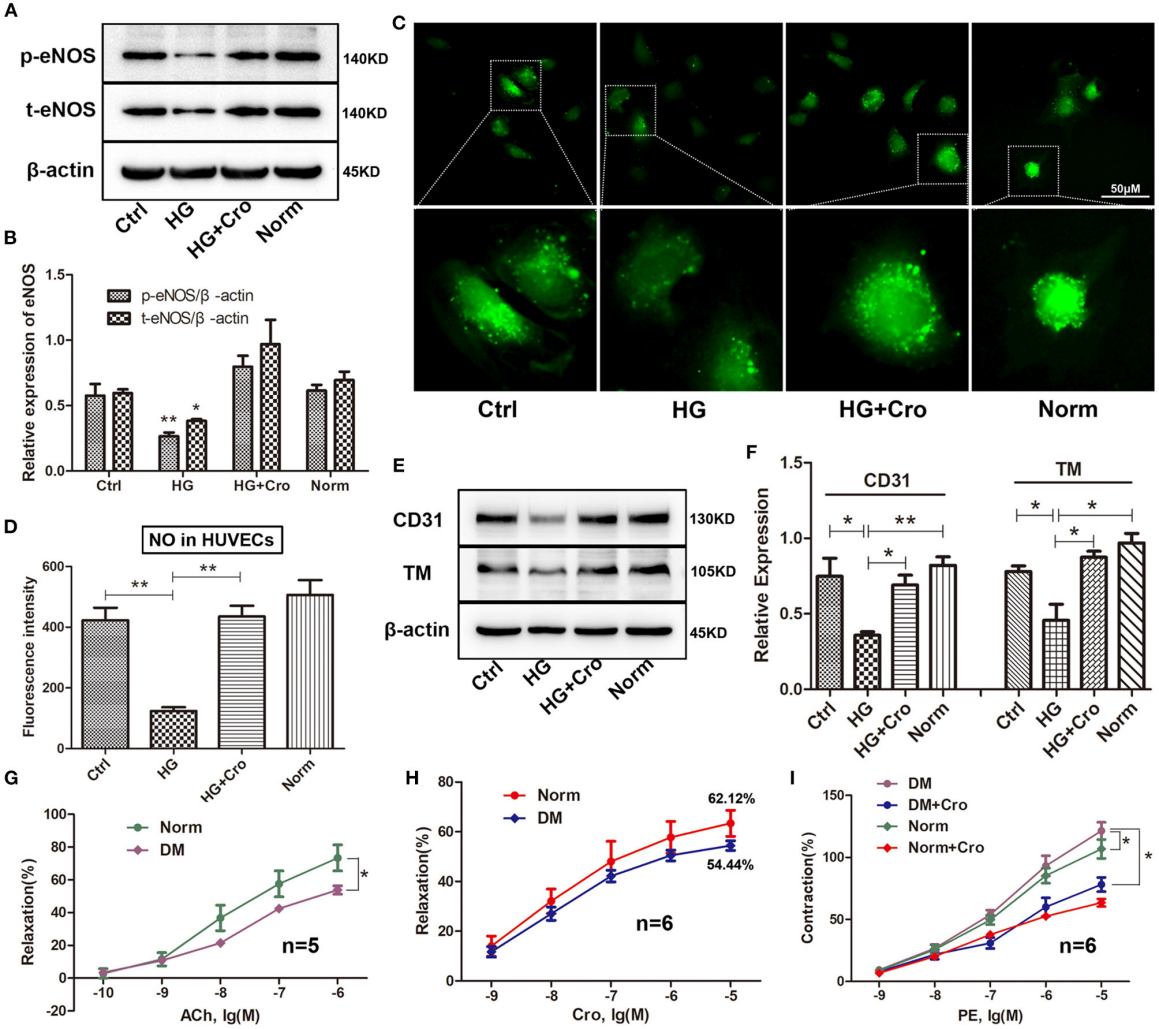
Crocin improves the hyperglycemia-induced endothelial dysfunction. Crocin was used to detect the alterations in the expression of t-eNOS, p-eNOS, CD31, and TM, as well as NO generation in HG-cultured HUVECs. Further, a myograph study was executed to analyze vascular tone after crocin application in diabetic rats. Western blotting showed that crocin suppressed the HG-induced decreases of t-eNOS and p-eNOS **(A,B)**, as well as CD31 and TM **(E,F)**. **(C,D)** Fluorescence staining determined that crocin reversed the reduction of NO generation by HG. **(G)** Hyperglycemia reduced the ACh-induced relaxation of artery rings. **(H)** The maximum vasodilation of diabetic arterial ring was noticeably restored to 88% (relaxation ratio: 54.44/62.12) of the physiological level after crocin treatment. **(I)** Hyperglycemia elevated the contractive effect of PE on artery rings, while this alteration was remarkably suppressed after pretreatment with crocin for 30 min. Ctrl, control; HG, high glucose; Cro, crocin; Norm, normal glucose; DM, diabetes mellitus; PE, phenylephrine; HUVECs, human umbilical vein endothelial cells; NO, nitric oxide; TM, thrombomodulin; eNOS, endothelial nitric oxide synthase; ACh, acetylcholine; PE, phenylephrine. Myograph data are means ± SEM from five to six different animals and analyzed using unpaired two-tailed Student's *t*-test and one-way ANOVA followed by Tukey's multiple comparisons test. ^*^p < 0.05, ^**^p < 0.01.

Crocin (10 μmol/L) was used to test the changes in t-/p-eNOS expression and NO production in HG-cultured HUVECs *in vitro*. Our data showed that crocin could significantly counteract the decrease in t-eNOS and p-eNOS expression induced by HG (Figures 6A,B) and in turn enhance NO generation (Figures 6C,D). In addition, Western blotting analysis revealed that crocin could prevent CD31 and TM from being downregulated by HG (Figures 6E,F). Further myograph experiments were performed to explore the effect of crocin on the vascular tone of artery rings in diabetic rats. The results showed that hyperglycemia damaged the vascular vessels, leading to a weakened response of aortic rings to ACh and poor vascular relaxation (Figure 6G). As shown in Figure 6H, the maximum vasodilation of the diabetic arterial ring was noticeably restored to 88% of the physiological level after crocin treatment (10^−5^ mol/L). We then confirmed that hyperglycemia elevated the contractive effect of PE on artery rings, resulting in an enhancement of vascular tone (Figure 6I). Interestingly, our data illustrated that when the diabetic artery rings were pretreated with crocin for 30 min, the contractive responses of artery rings to PE decreased remarkably close to normal levels (Figure 6I). These findings reveal that crocin could significantly improve hyperglycemia-induced endothelial dysfunction.

## Discussion

Diabetes-induced endothelial dysfunction is a crucial event that initiates the biological processes associated with diabetic vascular complications (Kaur et al., 2018; Maamoun et al., 2019). Previous studies have shown that crocin protects against endothelial damage through inhibiting apoptosis of endothelial cells (Xu et al., 2007; Razavi et al., 2016). Furthermore, our prior research has also indicated that crocin dramatically improves endothelial function, in which KCa3.1-modulated NO production and intracellular Ca^2+^ dynamics appear to be central mechanisms (Yang et al., 2018). In this study, we explored the potential protective effect of crocin against endothelial dysfunction through its mitochondrial protective effect in diabetes mellitus.

Generally, some events, such as excessive ROS generation, increased oxidative stress and enhanced the production of inflammatory factors, contributing to endothelial dysfunction (Sena et al., 2013; Shi and Vanhoutte, 2017). Mitochondria are a dominant source of cellular ROS in tissue (Dan Dunn et al., 2015), and hyperglycemia-induced mitochondrial dysfunction is a dominant cause of excessive ROS production in diabetes (Widlansky and Hill, 2018). Our data showed that crocin could significantly repress the HG-induced increase in ROS in a slightly dose-dependent manner, so it is justifiably hypothesized that mitochondria are an additional possible target of crocin in antioxidant effects. Hence, mitochondrial function was analyzed to evaluate the antioxidant mechanism of crocin through the MMP, mitochondrial mass, and mitochondrial morphology tests in this study. Our evidence indicated that high levels of glucose (33 mmol/L) to a great extent dissipated the MMP and mitochondrial mass and induced serious apoptosis and mitochondrial damage characterized by fragmented cristae, swelling, and vacuole formation. Intriguingly, our data showed that crocin could significantly reverse these pathological alterations and even promote mitochondrial fusion (Figure 1G). Mitochondrial mass (related to the number of mitochondria to some extent) is also a surrogate metabolic biomarker of mitochondrial biogenesis (Lamb et al., 2015). In addition, mitochondrial fusion and fission are two crucial events of mitochondrial dynamics driving mitochondrial metabolism (Wada and Nakatsuka, 2016). Among them, mitochondrial fusion, as the process by which two mitochondria merge in structure, is beneficial to the repair of damaged mitochondria *via* the exchange of material between damaged and non-damaged mitochondria (Wada and Nakatsuka, 2016). However, some disorders, such as diabetes mellitus, generally impair mitochondrial fusion (Wada and Nakatsuka, 2016; Rovira-Llopis et al., 2017). Therefore, our findings indicated that crocin could significantly prevent the HG-induced mitochondrial dysfunction. In particular, the mitochondrial fusion in TEM images strongly indicates the protective effect of crocin on mitochondrial function. Moreover, a previous paper confirmed that dysfunctional mitochondria could generate excessive ROS, which was closely connected to endothelial dysfunction in diabetes (Widlansky and Hill, 2018). Hence, the improvement of mitochondrial function might be a mechanism by which crocin effectively attenuates excessive ROS production in HG-cultured HUVECs. In other words, crocin may function as a mitochondria-targeted antioxidant in diabetes to a great extent.

In mitochondria, the K^+^ cycle maintains volume homeostasis *via* K^+^ channels by preventing excessive mitochondrial matrix swelling and contraction, which mediates mitochondrial function and ROS production (Garlid and Paucek, 2003). We first used confocal microscopy to identify the location of KCa3.1 channels in mitochondria. As a kind of K^+^ channel, mitochondrial KCa3.1 exerts K^+^ influx resulting in an increase in mitochondrial volume and depolarization (Garlid and Paucek, 2003). These data make it tempting to hypothesize the possible relevance of KCa3.1 to mitochondrial function. As expected, blockade of KCa3.1 by TRAM-34 could significantly reverse the HG-reduced MMP and mitochondrial mass and further repress excessive ROS production. Furthermore, KCa3.1 deficiency induced by siRNA also significantly reduced the overproduction of ROS and 4HNE (a kind of end product of lipid peroxidation), which is similar to the effect of crocin. Further study confirmed that the blockade and/or activation as well as deficiency of KCa3.1 could not affect the expression of NOX2 and SOD1, suggesting that mitochondria might be a dominant system by which KCa3.1 regulates ROS generation in diabetes. Concerning these present observations, we propose that long-term and/or severe hyperglycemic diabetes dramatically induces a compensatory increase in mitochondrial KCa3.1 expression levels, and in turn, this alteration results in pernicious K^+^ influx into the mitochondrial matrix, which leads subsequently to mitochondrial swelling and MMP dissipation. Therefore, KCa3.1-modulated mitochondrial volume homeostasis and the MMP are strongly proposed as additional central pathways that maintain mitochondrial function.

Notably, KCa3.1 has already been considered a vital target in a variety of cardiovascular diseases correlated with endothelial dysfunction (Feletou, 2009; Mathew John et al., 2018). In diabetes, KCa3.1-modulated endothelium-dependent hyperpolarization functions as a primary compensation mechanism for endothelial dysfunction (Shi and Vanhoutte, 2017). Given this mechanism, the alteration of KCa3.1 expression was investigated in diabetic mice and HG-cultured HUVECs in this job. As expected, our findings revealed that hyperglycemia and/or HG dramatically increased KCa3.1 expression in endothelial cells and even smooth muscle cells in vessels. However, different studies have demonstrated several controversies about the expressions of KCa3.1, indicating that it decreased (Weston et al., 2008; Zhao et al., 2014), remained unchanged (Ding et al., 2005), or even increased (Leo et al., 2011; Huang et al., 2014) in diabetes. Among them, some reports indicated that the elevated expression of KCa3.1 was a compensatory response due to the partial loss of KCa3.1 channel function induced by severe diabetes (duration of diabetes >15 weeks and/or hyperglycemia >10 mmol/L) (Goto and Kitazono, 2019). This statement is consistent with our result showing that endothelial KCa3.1 expression was enhanced by high levels of glucose (33 mmol/L) in cultured cells and/or hyperglycemia (16.7 mmol/L) in diabetic mice. We assume that ROS overproduction might be the cause of elevated KCa3.1 expression in diabetes. As expected, our subsequent study strongly proved this hypothesis that ROS from Rosup induced a significant increase in KCa3.1 expression, which was counteracted by NAC, a ROS scavenger (Figures 4B,C). These data suggest that KCa3.1 might be a key regulatory target of ROS in diabetes, which is also supported by a previous report that H_2_O_2_ increased KCa3.1 expression (Choi et al., 2013). Moreover, crocin significantly suppressed excessive ROS generation and KCa3.1 expression elevation induced by hyperglycemia *in vitro* and *in vivo*. Hence, it is possible that crocin attenuates excessive ROS production to restore or maintain the normal stage of KCa3.1 (expression and/or activity), which contributes to protecting mitochondrial function and subsequently reducing ROS production. In addition to NOX and SOD, GPx is another central member of the enzyme system that generally maintains the homeostasis of ROS under physiological conditions. GPx1, which is localized in the cytosol and mitochondria, reduces ROS levels by principally transforming hydrogen peroxide (H_2_O_2_) into water (H_2_O) in the body (He et al., 2017). Interestingly, crocin could significantly promote GPx1 expression and activation, suggesting that elimination of H_2_O_2_ is a crucial event in the process by which crocin reduces excessive ROS in diabetes. Hence, the GPx1/ROS (H_2_O_2_)/KCa3.1/mitochondrion pathway may be a regulatory axis of crocin in its antioxidant effect.

Several pieces of evidence have previously demonstrated that extracellular ROS can trigger rapid Ca^2+^ mobilization, which in turn enhances mitochondrial ROS production in endothelial cells (Hool and Corry, 2007; Feissner et al., 2009). Indeed, coupled with its known energy metabolism, mitochondria also serve as a very efficient Ca^2+^ buffer to take up substantial amounts of cytosolic Ca^2+^ (Feissner et al., 2009). Under the driving force derived from the MMP, moderate Ca^2+^ generally enters the mitochondrial matrix *via* the mitochondrial calcium uniporter (MCU) to modulate mitochondrial metabolism (Nicholls, 2005; Bravo-Sagua et al., 2017). However, excessive Ca^2+^ entry into mitochondria leads to Ca^2+^ overload, which results in mitochondrial dysfunction and even severe structural damage, including dissipation of the MMP, enhanced ROS formation, and increased mitochondrial fragmentation (Ohshima et al., 2017). Meanwhile, increased Ca^2+^ in turn adequately activates mitochondrial KCa3.1 channels, leading to drastic K^+^ influx into the mitochondrial matrix (Kang et al., 2007). Intriguingly, crocin significantly restrained the intracellular Ca^2+^ increase in this study. Therefore, Ca^2+^ may be a crucial modulator when crocin protects against mitochondrial dysfunction. In addition, crocin significantly reversed these HG-decreased factors, such as t-eNOS, p-eNOS, CD31, and TM, as well as NO production in endothelial cells. Crocin also noticeably prevented diabetes-damaged vascular tone in the artery in a myograph study. These abnormal alterations of NO release, CD31 and TM expression, and vascular tone, to some extent, are central responses to endothelial dysfunction (Hasibuzzaman et al., 2017; Manetti et al., 2017; Shi and Vanhoutte, 2017). Therefore, these data indicate that crocin can effectively improve the endothelial dysfunction induced by hyperglycemia. However, crocin did not improve elevated fasting blood glucose levels or body weight alterations in diabetic mice (data not shown), suggesting that crocin could not effectively control blood glucose levels in diabetes.

**Figure 7:**
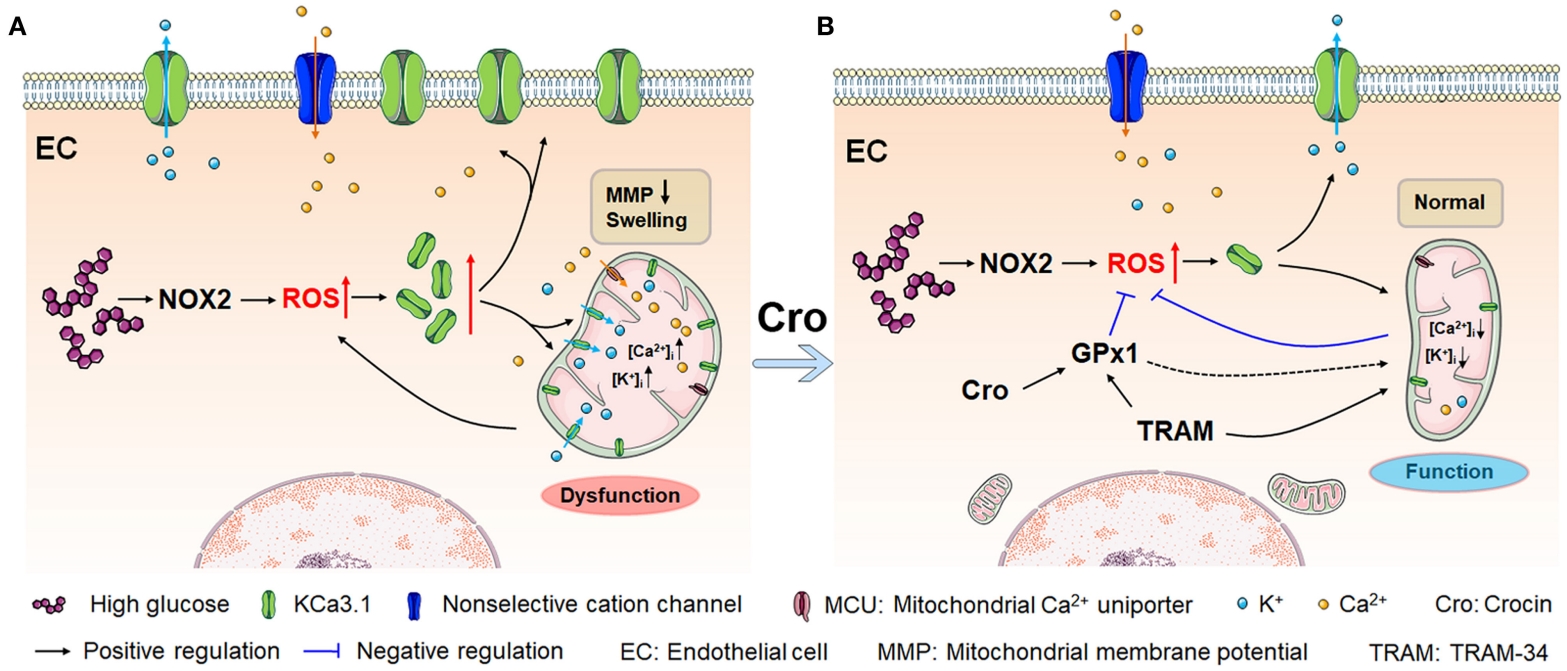
Crocin counteracts hyperglycemia-induced ROS by improving mitochondrial function regulated by KCa3.1. **(A)** Hyperglycemia/NOX2-induced excessive ROS enhances KCa3.1 expression, which is triggered by abundant mitochondrial Ca^2+^, leading to dramatic K^+^ influx into the mitochondria. This process subsequently induces severe mitochondrial swelling, fragmented cristae, and dysfunction, secondarily resulting in more ROS generation. This vicious cycle forms an underlying mechanism that leads to excessive ROS production in diabetes, in which KCa3.1 plays a pivotal role. **(B)** Crocin reverses the vicious cycle to prevent the mitochondrial dysfunction by inhibiting the ROS-induced KCa3.1 expression elevation, which implies that crocin is a potential mitochondria-targeted antioxidant. Here, KCa3.1-modulated mitochondrial volume homeostasis might play a central role in ROS generation. ROS, reactive oxygen species; NOX2, NADPH oxidase 2.

Conclusively, as presented in Figure 7A, HG and/or hyperglycemia in diabetes mellitus promotes ROS overproduction by enhancing NOX2 in endothelial cells, which leads to a dramatic rise in KCa3.1 expression in the endothelium. (1) In the plasma membrane, KCa3.1 expression elevation increases intracellular Ca^2+^ levels, which forms a high transmembrane electrochemical gradient of Ca^2+^ in mitochondria. Additionally, excessive ROS promotes rapid entry of Ca^2+^ in the cytoplasm into mitochondria *via* MCU carriers, leading to severe Ca^2+^ overload. (2) In mitochondria, increased KCa3.1 expression promotes dramatic K^+^ influx into the mitochondrial matrix, resulting in subsequent mitochondrial swelling. These two events, namely, Ca^2+^ overload and mitochondrial swelling due to the elevation of KCa3.1 expression, could ultimately lead to mitochondrial dysfunction and subsequent ROS overproduction, and in turn, excessive ROS would enhance the expression of KCa3.1. Hence, a vicious cycle seems to be formed between excessive ROS generation and mitochondrial dysfunction, wherein KCa3.1 serves as a pivotal player by modulating K^+^ and Ca^2+^ mobilization. This is a novel possible mechanism illuminated by our study in which NOX2-originated ROS in turn induces more ROS generation. Intriguingly, as shown in Figure 7B, crocin possibly counteracts ROS-enhanced KCa3.1 expression by elevating GPx1. Subsequently, inhibition of KCa3.1 could prevent mitochondrial dysfunction by decreasing excessive K^+^ influx into the mitochondrion, which in turn leads to a decrease in ROS generation. These results reveal a virtuous cycle of ROS generation due to crocin treatment. In summary, crocin improved diabetes-induced endothelial dysfunction by protecting mitochondrial function and decreasing subsequent ROS overproduction, in which KCa3.1 is a central modulator to maintain mitochondrial volume homeostasis.

## Data Availability Statement

The original contributions presented in the study are included in the article/supplementary material, further inquiries can be directed to the corresponding authors.

## Ethics Statement

The animal study was reviewed and approved by the Animal Care and Use Committee of Harbin Medical University and the Care and Use of Laboratory Animals (US National Institutes of Health).

## Author Contributions

HY designed and supervised this study. YZ was involved in the designing experiment. XuL, YL, CLi, LW, XLi, QW, and XD carried out the experiments. AC, XLv, JZ, HC, and CLa conducted data analyses. XuL and YL drafted the manuscript. All authors contributed to and have approved the final manuscript.

## Conflict of Interest

The authors declare that the research was conducted in the absence of any commercial or financial relationships that could be construed as a potential conflict of interest.

## References

[B1] Bravo-Sagua R. Parra V. Lopez-Crisosto C. Diaz P. Quest A. F. Lavandero S. (2017). Calcium transport and signaling in mitochondria. Compr. Physiol. 7, 623–634. 10.1002/cphy.c160013 28333383

[B2] Choi S. Na H. Y. Kim J. A. Cho S. E. Suh S. H. (2013). Contradictory effects of superoxide and hydrogen peroxide on KCa3.1 in human endothelial cells. Korean J. Physiol. Pharmacol. 17, 181–187. 10.4196/kjpp.2013.17.3.181 23776393 PMC3682077

[B3] Chung B. H. Kim S. Kim J. D. Lee J. J. Baek Y. Y. Jeoung D.. (2012). Syringaresinol causes vasorelaxation by elevating nitric oxide production through the phosphorylation and dimerization of endothelial nitric oxide synthase. Exp. Mol. Med. 44, 191–201. 10.3858/emm.2012.44.3.014 22170035 PMC3317483

[B4] Damkjaer M. Nielsen G. Bodendiek S. Staehr M. Gramsbergen J. B. de Wit C.. (2012). Pharmacological activation of KCa3.1/KCa2.3 channels produces endothelial hyperpolarization and lowers blood pressure in conscious dogs. Br. J. Pharmacol. 165, 223–234. 10.1111/j.1476-5381.2011.01546.x 21699504 PMC3252979

[B5] Dan Dunn J. Alvarez L. A. Zhang X. Soldati T. (2015). Reactive oxygen species and mitochondria: a nexus of cellular homeostasis. Redox Biol. 6, 472–485. 10.1016/j.redox.2015.09.005 26432659 PMC4596921

[B6] Deanfield J. E. Halcox J. P. Rabelink T. J. (2007). Endothelial function and dysfunction: testing and clinical relevance. Circulation 115, 1285–1295. 10.1161/CIRCULATIONAHA.106.652859 17353456

[B7] Ding H. Hashem M. Wiehler W. B. Lau W. Martin J. Reid J.. (2005). Endothelial dysfunction in the streptozotocin-induced diabetic apoE-deficient mouse. Br. J. Pharmacol. 146, 1110–1118. 10.1038/sj.bjp.0706417 16231005 PMC1751246

[B8] Fagot D. Pham D. M. Laboureau J. Planel E. Guerin L. Negre C.. (2018). Crocin, a natural molecule with potentially beneficial effects against skin aging. Int. J. Cosmet. Sci. 40, 388–400. 10.1111/ics.12472 29893408

[B9] Favero G. Paganelli C. Buffoli B. Rodella L. F. Rezzani R. (2014). Endothelium and its alterations in cardiovascular diseases: life style intervention. BioMed. Res. Int. 2014:801896. 10.1155/2014/801896 24719887 PMC3955677

[B10] Feissner R. F. Skalska J. Gaum W. E. Sheu S. S. (2009). Crosstalk signaling between mitochondrial Ca2+ and ROS. Front. Biosci. 14, 1197–1218. 10.2741/3303 19273125 PMC2683671

[B11] Feletou M. (2009). Calcium-activated potassium channels and endothelial dysfunction: therapeutic options? Br. J. Pharmacol. 156, 545–562. 10.1111/j.1476-5381.2009.00052.x 19187341 PMC2697708

[B12] Garlid K. D. Paucek P. (2003). Mitochondrial potassium transport: the K(+) cycle. Biochim. Biophys. Acta 1606, 23–41. 10.1016/S0005-2728(03)00108-7 14507425

[B13] Ghorbanzadeh V. Mohammadi M. Mohaddes G. Dariushnejad H. Chodari L. Mohammadi S. (2016). Protective effect of crocin and voluntary exercise against oxidative stress in the heart of high-fat diet-induced type 2 diabetic rats. Physiol. Int. 103, 459–468. 10.1556/2060.103.2016.4.6 28229629

[B14] Goto K. Kitazono T. (2019). Endothelium-dependent hyperpolarization (EDH) in diabetes: mechanistic insights and therapeutic implications. Int. J. Mol. Sci. 20:3737. 10.3390/ijms20153737 31370156 PMC6695796

[B15] Grgic I. Kaistha B. P. Hoyer J. Kohler R. (2009). Endothelial Ca+-activated K+ channels in normal and impaired EDHF-dilator responses–relevance to cardiovascular pathologies and drug discovery. Br. J. Pharmacol. 157, 509–526. 10.1111/j.1476-5381.2009.00132.x 19302590 PMC2707963

[B16] Hasibuzzaman M. M. Hossain S. Islam M. S. Rahman A. Anjum A. Hossain F.. (2017). Association between arsenic exposure and soluble thrombomodulin: a cross sectional study in Bangladesh. PloS ONE 12:e0175154. 10.1371/journal.pone.0175154 28399171 PMC5388467

[B17] He L. He T. Farrar S. Ji L. Liu T. Ma X. (2017). Antioxidants maintain cellular redox homeostasis by elimination of reactive oxygen species. Cell. Physiol. Biochem. 44, 532–553. 10.1159/000485089 29145191

[B18] Hool L. C. Corry B. (2007). Redox control of calcium channels: from mechanisms to therapeutic opportunities. Antioxid. Redox Signal. 9, 409–435. 10.1089/ars.2006.1446 17280484

[B19] Huang C. Pollock C. A. Chen X. M. (2014). Role of the potassium channel KCa3.1 in diabetic nephropathy. Clin. Sci. 127, 423–433. 10.1042/CS20140075 24963668

[B20] Huang C. Shen S. Ma Q. Chen J. Gill A. Pollock C. A.. (2013). Blockade of KCa3.1 ameliorates renal fibrosis through the TGF-beta1/Smad pathway in diabetic mice. Diabetes 62, 2923–2934. 10.2337/db13-0135 23656889 PMC3717839

[B21] Huang C. Zhang L. Shi Y. Yi H. Zhao Y. Chen J.. (2018). The KCa3.1 blocker TRAM34 reverses renal damage in a mouse model of established diabetic nephropathy. PloS ONE 13:e0192800. 10.1371/journal.pone.0192800 29425253 PMC5806905

[B22] Kang S. H. Park W. S. Kim N. Youm J. B. Warda M. Ko J. H.. (2007). Mitochondrial Ca2+-activated K+ channels more efficiently reduce mitochondrial Ca2+ overload in rat ventricular myocytes. Am. J. Physiol. Heart Circ. Physiol. 293, H307–H313. 10.1152/ajpheart.00789.2006 17351070

[B23] Kaur R. Kaur M. Singh J. (2018). Endothelial dysfunction and platelet hyperactivity in type 2 diabetes mellitus: molecular insights and therapeutic strategies. Cardiovasc. Diabetol. 17:121. 10.1186/s12933-018-0763-3 30170601 PMC6117983

[B24] King A. J. (2012). The use of animal models in diabetes research. Br. J. Pharmacol. 166, 877–894. 10.1111/j.1476-5381.2012.01911.x 22352879 PMC3417415

[B25] Klumpp L. Sezgin E. C. Skardelly M. Eckert F. Huber S. M. (2018). KCa3.1 channels and glioblastoma: *in vitro* studies. Curr. Neuropharmacol. 16, 627–635. 10.2174/1570159X15666170808115821 28786347 PMC5997865

[B26] Lamb R. Bonuccelli G. Ozsvari B. Peiris-Pages M. Fiorillo M. Smith D. L.. (2015). Mitochondrial mass, a new metabolic biomarker for stem-like cancer cells: understanding WNT/FGF-driven anabolic signaling. Oncotarget 6, 30453–30471. 10.18632/oncotarget.5852 26421711 PMC4741544

[B27] Leanza L. O'Reilly P. Doyle A. Venturini E. Zoratti M. Szegezdi E.. (2014). Correlation between potassium channel expression and sensitivity to drug-induced cell death in tumor cell lines. Curr. Pharm. Des. 20, 189–200. 10.2174/13816128113199990032 23701546

[B28] Leo C. H. Hart J. L. Woodman O. L. (2011). Impairment of both nitric oxide-mediated and EDHF-type relaxation in small mesenteric arteries from rats with streptozotocin-induced diabetes. Br. J. Pharmacol. 162, 365–377. 10.1111/j.1476-5381.2010.01023.x 20840539 PMC3031058

[B29] Liu W. Sun Y. Cheng Z. Guo Y. Liu P. Wen Y. (2018). Crocin exerts anti-inflammatory and anti-arthritic effects on type II collagen-induced arthritis in rats. Pharm. Biol. 56, 209–216. 10.1080/13880209.2018.1448874 29540097 PMC6168764

[B30] Maamoun H. Abdelsalam S. S. Zeidan A. Korashy H. M. Agouni A. (2019). Endoplasmic reticulum stress: a critical molecular driver of endothelial dysfunction and cardiovascular disturbances associated with diabetes. Int. J. Mol. Sci. 20:1658. 10.3390/ijms20071658 30987118 PMC6480154

[B31] Manetti M. Romano E. Rosa I. Guiducci S. Bellando-Randone S. De Paulis A.. (2017). Endothelial-to-mesenchymal transition contributes to endothelial dysfunction and dermal fibrosis in systemic sclerosis. Ann. Rheum. Dis. 76, 924–934. 10.1136/annrheumdis-2016-210229 28062404

[B32] Mathew John C. Khaddaj Mallat R. George G. Kim T. Mishra R. C. Braun A. P. (2018). Pharmacologic targeting of endothelial Ca(2+)-activated K(+) channels: a strategy to improve cardiovascular function. Channels 12, 126–136. 10.1080/19336950.2018.1454814 29577810 PMC5972810

[B33] McLaughlin K. Audette M. C. Parker J. D. Kingdom J. C. (2018). Mechanisms and clinical significance of endothelial dysfunction in high-risk pregnancies. Can. J. Cardiol. 34, 371–380. 10.1016/j.cjca.2018.01.006 29571421

[B34] Nicholls D. G. (2005). Mitochondria and calcium signaling. Cell Calcium 38, 311–317. 10.1016/j.ceca.2005.06.011 16087232

[B35] Ohshima Y. Takata N. Suzuki-Karasaki M. Yoshida Y. Tokuhashi Y. Suzuki-Karasaki Y. (2017). Disrupting mitochondrial Ca2+ homeostasis causes tumor-selective TRAIL sensitization through mitochondrial network abnormalities. Int. J. Oncol. 51, 1146–1158. 10.3892/ijo.2017.4096 28849210

[B36] Rajendran P. Rengarajan T. Thangavel J. Nishigaki Y. Sakthisekaran D. Sethi G.. (2013). The vascular endothelium and human diseases. Int. J. Biol. Sci. 9, 1057–1069. 10.7150/ijbs.7502 24250251 PMC3831119

[B37] Razavi B. M. Hosseinzadeh H. Abnous K. Khoei A. Imenshahidi M. (2016). Protective effect of crocin against apoptosis induced by subchronic exposure of the rat vascular system to diazinon. Toxicol. Ind. Health 32, 1237–1245. 10.1177/0748233714554941 27353299

[B38] Rovira-Llopis S. Banuls C. Diaz-Morales N. Hernandez-Mijares A. Rocha M. Victor V. M. (2017). Mitochondrial dynamics in type 2 diabetes: pathophysiological implications. Redox Biol. 11, 637–645. 10.1016/j.redox.2017.01.013 28131082 PMC5284490

[B39] Sena C. M. Pereira A. M. Seica R. (2013). Endothelial dysfunction - a major mediator of diabetic vascular disease. Biochim. Biophys. Acta 1832, 2216–2231. 10.1016/j.bbadis.2013.08.006 23994612

[B40] Shi Y. Vanhoutte P. M. (2017). Macro- and microvascular endothelial dysfunction in diabetes. J. Diabetes 9, 434–449. 10.1111/1753-0407.12521 28044409

[B41] Su X. L. Zhang H. Yu W. Wang S. Zhu W. J. (2013). Role of KCa3.1 channels in proliferation and migration of vascular smooth muscle cells by diabetic rat serum. Chin. J. Physiol. 56, 155–162. 10.4077/cjp.2013.bab104 23656217

[B42] Wada J. Nakatsuka A. (2016). Mitochondrial dynamics and mitochondrial dysfunction in diabetes. Acta Med. Okayama 70, 151–158. 10.18926/AMO/54413 27339203

[B43] Weston A. H. Absi M. Harno E. Geraghty A. R. Ward D. T. Ruat M.. (2008). The expression and function of Ca(2+)-sensing receptors in rat mesenteric artery; comparative studies using a model of type II diabetes. Br. J. Pharmacol. 154, 652–662. 10.1038/bjp.2008.108 18414396 PMC2439515

[B44] Widlansky M. E. Hill R. B. (2018). Mitochondrial regulation of diabetic vascular disease: an emerging opportunity. Transl. Res. 202, 83–98. 10.1016/j.trsl.2018.07.015 30144425 PMC6218302

[B45] Widmer R. J. Lerman A. (2014). Endothelial dysfunction and cardiovascular disease. Glob. Cardiol. Sci. Pract. 2014, 291–308. 10.5339/gcsp.2014.43 25780786 PMC4352682

[B46] Wolfle S. E. Schmidt V. J. Hoyer J. Kohler R. de Wit C. (2009). Prominent role of KCa3.1 in endothelium-derived hyperpolarizing factor-type dilations and conducted responses in the microcirculation *in vivo*. Cardiovasc. Res. 82, 476–483. 10.1093/cvr/cvp060 19218287

[B47] Xu G. Gong Z. Yu W. Gao L. He S. Qian Z. (2007). Increased expression ratio of Bcl-2/Bax is associated with crocin-mediated apoptosis in bovine aortic endothelial cells. Basic Clin. Pharmacol. Toxicol. 100, 31–35. 10.1111/j.1742-7843.2007.00001.x 17214608

[B48] Xu G. Zhao X. Fu J. Wang X. (2019). Resveratrol increase myocardial Nrf2 expression in type 2 diabetic rats and alleviate myocardial ischemia/reperfusion injury (MIRI). Ann. Palliat. Med. 8, 565–575. 10.21037/apm.2019.11.25 31865720

[B49] Yang H. Li X. Liu Y. Li X. Li X. Wu M.. (2018). Crocin improves the endothelial function regulated by Kca3.1 through ERK and Akt signaling pathways. Cell. Physiol. Biochem. 46, 765–780. 10.1159/000488735 29621746

[B50] Yang H. Li X. Ma J. Lv X. Zhao S. Lang W.. (2013). Blockade of the intermediate-conductance Ca(2+)-activated K+ channel inhibits the angiogenesis induced by epidermal growth factor in the treatment of corneal alkali burn. Exp. Eye Res. 110, 76–87. 10.1016/j.exer.2013.02.015 23482085

[B51] Yang Q. Huang J. H. Yao X. Q. Underwood M. J. Yu C. M. (2014). Activation of canonical transient receptor potential channels preserves Ca2+ entry and endothelium-derived hyperpolarizing factor-mediated function *in vitro* in porcine coronary endothelial cells and coronary arteries under conditions of hyperkalemia. J. Thorac. Cardiovasc. Surg. 148, 1665–1673.e1. 10.1016/j.jtcvs.2014.02.026 24629221

[B52] Zhao L. M. Wang Y. Ma X. Z. Wang N. P. Deng X. L. (2014). Advanced glycation end products impair K(Ca)3.1- and K(Ca)2.3-mediated vasodilatation via oxidative stress in rat mesenteric arteries. Pflugers Arch. 466, 307–317. 10.1007/s00424-013-1324-y 23873353

